# Re-evaluating the Morse Fall Scale in obstetrics and gynecology wards and determining optimal cut-off scores for enhanced risk assessment: A retrospective survey

**DOI:** 10.1371/journal.pone.0305735

**Published:** 2024-09-05

**Authors:** Bijun Mao, Huiping Jiang, Yan Chen, Chunsheng Wang, Luping Liu, Huifeng Gu, Ya Shen, Peihong Zhou

**Affiliations:** 1 Nursing Department, Huzhou Maternity & Child Health Care Hospital, Huzhou, China; 2 School of Medicine, Huzhou Teachers College, Huzhou, China; King Saud University Medical City, SAUDI ARABIA

## Abstract

**Objective:**

This study aims to examine the validity of the MFS by analyzing the electronic medical records on fall risk in obstetrics and gynecology wards and determine the optimal cut-off score of the Morse Fall Scale.

**Design:**

A retrospective survey.

**Methods:**

The research was conducted in an Obstetrics and Gynecology Hospital and a general hospital. The sample included 136 fall inpatients and 120 no-fall inpatients recruited from January 1st, 2020, to July 10th, 2022. The Morse Fall Scale was analyzed using the gold standard of patients who fell while hospitalized, assessing the area under the Receiver Operating Characteristic curve, sensitivity, specificity, accuracy, positive predictive value, negative predictive value, and Kappa.

**Results:**

At cut-off scores of 40, 45,50, and 55, the area under the Receiver Operating Characteristic curve was 0.772, 0.761, 0.749, and 0.763, respectively. The Youden index was 0.543, 0.521, 0.498, and 0.525, while Kappa values were 0.540, 0.518, 0.490, and 0.515. Sensitivity was 0.735, 0.713, 0.640, and 0.625; specificity was 0.808, 0.808, 0.858, and 0.900. The positive predictive values were 0.813, 0.808, 0.837, and 0.876, and the negative predictive values were 0.729, 0.713, 0.678, and 0.679. Accuracy were 0.770, 0.758, 0.742, and 0.754.

**Conclusions:**

The Morse Fall Scale demonstrates good predictive performance for assessing fall risk in gynecology and obstetrics wards. The optimal cut-off score is 40.

## 1 Introduction

Falls pose a pervasive and significant threat to patient safety, particularly among inpatients. Statistics suggest that more than one million patients experience falls in hospitals annually [1]. The prevalence and impact of falls in hospital settings are widely acknowledged due to the potential for prolonged hospital stays, physical injuries, and, in severe cases, fatalities [2]. Globally, falls have emerged as a representative benchmark for assessing nursing quality [3]. Addressing and preventing accidental falls in patients consistently remains a critical aspect of safety and quality management.

A pivotal strategy in fall prevention involves the proactive identification of patients at risk of falling. Employing appropriate fall assessment tools is crucial in aiding nurses to promptly and accurately gauge the level of fall risk, facilitating the targeted implementation of preventive measures.

Currently, there exist several fall risk assessment tools designed to identify the risk of falls in patients. In numerous research studies involving diverse populations, validation has been established for tools such as the St Thomas’s Risk Assessment Tool in Falling Elderly Inpatients (STRATIFY), Morse Fall Scale (MFS), and Hendrich Fall Risk Model (HFRM) [4]. Despite these tools demonstrating moderate-to-good predictive validity and reliability across various studies, divergent views have been presented. A previous systematic review on fall-risk screening concluded that due to the multidimensional nature of fall risk, there is no universally "ideal" tool applicable in every context or capable of performing flawless risk assessments. While fall risk assessment tools demonstrated high accuracy in developmental settings, their effectiveness diminished in other care contexts, as suggested by Meekes et al [5]. Therefore, a reevaluation of the tools mentioned above is necessary when applied in different care contexts [6].

## 2 Background

The MFS was meticulously developed as a fall risk assessment tool through a rigorous design process by Morse, Black, et al. [4]. Comprising six scored items, the MFS assesses a patient’s likelihood of falling based on specific criteria: a history of falling (0 = No, 25 = Yes), the presence of a secondary diagnosis (0 = No, 15 = Yes), the use of ambulatory aids like a cane, wheelchair, or walking frame (0 = None, 15 = wheelchair/bed rest, 30 = nurse assist), the administration of intravenous therapy (0 = No, 20 = Yes), types of gait (0 = normal, 10 = bed rest, 20 = immobile), and mental status (0 = oriented to own ability, 15 = cannot orient to own ability). The scores on the MFS range from 0 to 125, with higher scores indicating a greater likelihood of falling [5]. According to Morse, the MFS demonstrated a sensitivity of 78%, a positive predictive value (PPV) of 10.3%, a specificity of 83%, and a high negative predictive value (NPV) of 99.2% [5]. Morse recommended a cut-off score of 45 for optimal use in long-term care wards, chronic disease wards, and emergency wards [5].

While the MFS has demonstrated accuracy in assessing fall risk within its developmental environment, scholars have observed variations in its performance across different patient groups [6,7]. According to Young Ju Kim [8], in an acute care setting, the MFS exhibited a sensitivity of 85.7% and a specificity of 58.8% when the cut-off score was set at 50. However, studies by Urbanetto reported that the Brazilian version of the MFS (MFS-B) exhibited only moderate reliability in predicting a patient’s risk of falling [9]. The best estimate to predict falls was found to be at the cut-off score of 44.78 for the average MFS-B score, with a sensitivity of 95.2% and a specificity of 64%. These findings suggest that the MFS performance varies across scenarios, emphasizing the need to adjust the cut-off score to align with specific patient populations and care contexts. Therefore, a reevaluation of MFS is necessary when applied in different care settings.

Currently, the MFS is extensively utilized within Chinese hospitals, particularly in obstetrics and gynecology wards. However, literature regarding its application in this specific setting remains scarce, with most studies focusing on emergency, surgical, and rehabilitation wards [5,6,8]. In the study conducted by Morse and her colleagues, the MFS was developed across various units within two institutions. These units included six from the acute care division (comprising general surgical, ophthalmology, and three medical units), two from the long-term care division (psycho-geriatric and nursing home), and eight adult units from a rehabilitation hospital. Notably, none of these units encompassed obstetrics and gynecology departments.

It is essential to recognize obstetrics and gynecology as a distinct discipline separate from internal medicine and surgery. Patients within this specialty are exclusively female, with care centered on reproductive and gynecological concerns. Surgical interventions commonly performed include extensive hysterectomy, curettage, cesarean section, laparoscopic surgery, management of menstrual disorders, miscarriage prevention, infertility, gynecological infections, and childbirth assistance.

Typically, obstetrics and gynecology inpatients are under 65 years old and in the reproductive stage, exhibiting superior muscle strength and physiological functionality compared to elderly patients. Obstetrics and gynecology inpatients possess unique characteristics compared to those in rehabilitation facilities and geriatric wards where the MFS was initially developed and tested. Therefore, concerns have been raised about the applicability of MFS in obstetrics and gynecology wards. These considerations prompt us to question, how effective is MFS in obstetrics and gynecology wards. whether the optimal cut-off score for the MFS in obstetrics and gynecology wards would deviate from the scores reported in previous studies. Therefore, it is very urgent to re-evaluate the effectiveness of MFS in obstetrics and gynecology wards and determine its optimal cut-off value to ensure the maximum utility of MFS application in obstetrics and gynecology wards.

The primary aim of this study is to examine the validity of the MFS in the context of obstetrics and gynecology wards. Additionally, to determine the optimal cut-off score for the MFS. This determination will be accomplished through a thorough analysis of essential metrics, including sensitivity, specificity, accuracy, the area under the Receiver Operating Characteristic curve (AUC), Youden index, Positive Predictive Value (PPV), Negative Predictive Value (NPV), and the Kappa index.

## 3 Methods

### 3.1. Aim

To examine the validity of the MFS by analyzing the electronic medical records on fall risk in obstetrics and gynecology wards and determine the optimal cut-off score of MFS.

### 3.2 Study design

This study was a retrospective survey designed, it was conducted from September to December 2022.

### 3.3 Definition of falls

A patient fall is defined as an unplanned descent to the ground, with or without resulting injury [10]. Our assessment includes various fall scenarios, incorporating assisted falls (pre-falls), where the descent is mitigated by prompt intervention from nurses or caregivers, thereby reducing the likelihood of injury, whether due to physiological or environmental factors.

### 3.4 Sample size calculation

Following Yung Hee Sung [11], who suggested 84 subjects for each group, we calculated the sample size using the formula: n = (Zα/δ)^2^p*(1-p) budget sample size, where α = 0.05, Zα = 1.96, and δ = 0.075. According to our pre-test, sensitivity (pse = 0.75), specificity (psp = 0.78), and the sample size were calculated as follows: 117 cases for the no-fall group and 128 for the fall group.

### 3.5. Setting and participants

This study was conducted in an Obstetrics and Gynecology hospital and a general hospital, encompassing 8 obstetrical wards and 7 gynecological wards(did not encompass labor rooms or delivery units), during the period from January 1st, 2020, to July 10th, 2022. The average duration of stay in these wards was 4–5 days.

A total of 63,568 patients met the inclusion criteria, comprising 136 fallers and 63,432 no-fallers. Fallers meeting the criteria were designated as the fall group. Each no-faller was assigned a sequential number, and then randomly selected 121 patients from this group using SPSS for inclusion in the control group. 1 patient was excluded from the analysis due to insufficient data, leaving 120 no-fall patients ultimately included in this study.

### 3.6. Data collection

In this study, we collected data on the incidence of patient falls during hospitalization, extracting records from the hospital’s adverse-event reporting system. Demographic details were obtained from admission records, and clinical information was retrieved from the hospital’s electronic database.

To prevent patient falls, the hospitals in this study routinely conducted fall risk assessments using the MFS. Nurses performed the initial assessment upon admission, revisiting it if there were changes in the patient’s medical condition. Nurses implemented preventive measures based on the MFS scores. For patients who experienced falls, we collected the MFS scores for the day of the fall event. For patients who did not experience falls, we recorded the maximum MFS score during the entire hospitalization period, as a higher MFS score indicates an elevated risk of falling.

### 3.7. Data analysis

The data were processed using SPSS for Windows, version 12.0.1(SPSS Inc., Chicago, IL, USA). Descriptive statistics, including frequencies, percentages, means, and standard deviations, were employed to analyze the demographic and clinical characteristics of the subjects. Relationships between subjects and demographic and clinical characteristics were assessed using χ2 tests and t-tests. Additionally, the t-test was utilized for normally distributed data, the Mann-Whitney U test for non-normally distributed data, and the chi-square test for comparing categorical data.

The optimal cut-off for the MFS was determined by analyzing its performance against the gold standard of patients who experienced falls during their hospitalization. This analysis included metrics such as the area under the Receiver Operating Characteristic curve (AUC), sensitivity, specificity, accuracy, Positive Predictive Value (PPV), Negative Predictive Value (NPV), and Kappa.

### 3.8. Ethical considerations

This research received approval from the ethics committee overseeing human subjects at the hospital where the study was conducted. All participants in this study were adults. To ensure confidentiality and privacy, the data file was encrypted and accessible exclusively to the principal researchers.

## 4 Results

### 4.1 Characteristics of subjects

A total of 136 inpatients who experienced falls and 120 inpatients who did not fall were included in this study. The cumulative inpatient days in the gynecology and obstetrics wards reached 269,533, resulting in a fall rate of 0.504 per 1000 patient days. Table 1 presents the characteristics of the participants, categorizing them based on their falling status. Notably, all participants were female, had no delirium, and there were no significant differences observed in falls among individuals in gynecological and obstetric wards concerning age, education, marital status, smoking, and drinking habits, inpatients who underwent surgery were more prone to falls (χ2 = 23.993, *P =* 0.000).

**Table 1:** Comparisons of characteristics between groups (*n* = 256, %).

		Fall (n = 136) n (%)/M ±SD	No-Fall (n = 120) n (%)/M ±SD	X^2^or t	*P-*value
**Age**	-	31.54±11.11	31.25±9.28	0.23	*0*.*820*
**Education**	Under the University	99(72.8)	96(80.0)	2.00	*0*.*177*
	University above	37(27.2)	24(20.0)		
**Marriage**	Divorcement	3(2.2)	2(1.7)	2.87	*0*.*455*
	The partner died	1(0.7)	0(0.0)		
	Unmarried	0(0.0)	2(1.7)		
	Married	132(97.1)	116(96.7)		
**Smoking**	Yes	1(0.7)	2(1.7)	0.48	*0*.*490*
	No	135(99.3)	118(98.3)		
**Drinking**	Yes	3(2.2)	4(3.3)	0.31	*0*.*581*
	No	133(97.8)	116(96.7)		
**Admission course**	Emergency	32(23.5)	26(21.7)	0.13	*0*.*722*
	Outpatient admission	104(76.5)	94(78.3)		
**Hospitalization (days)**	-	6.06±3.133	6.16±3.084	-0.26	*0*.*799*
**Admission method**	Walking	105(77.2)	102(85.0)	2.60	*0*.*273*
	Wheelchair	22(14.6)	12(10.0)		
	Stretcher car	9(6.6)	6(5.0)		
**Activity status**	Fully dependent	0(0.0)	0(0.0)	2.68	*0*.*102*
	Partially dependent	3(2.2)	0(0.0)		
	Independent	133(97.8)	120(100.0)		
**Diabetes**	Yes	20(14.7)	12(10.0)	1.29	*0*.*256*
	No	116(85.3)	108(90.0)		
**Hypertension**	Yes	20(14.7)	12(10.00)	1.29	*0*.*256*
	No	116(85.3)	108(90.0)		
**Insomnia**	Yes	8(5.9)	2(1.7)	3.02	*0*.*082*
	No	128(94.1)	118(98.3)		
**Surgery**	Yes	57(41.9)	31(25.8)	7.31	*0*.*007*
	No	79(58.1)	89(74.2)		
**Kidney disease**	Yes	0(0.0)	1(0.8)	1.14	*0*.*469*
	No	136(100.0)	119(99.2)		
**Hepatopathy**	Yes	3(2.2)	1 (0.8)	0.78	*0*.*377*
	No	133(97.8)	119 (99.2)		
**Mental sickness**	Yes	0(0.0)	1 (0.8)	1.14	*0*.*469*
	No	136(100.0)	119 (99.2)		
**Disease of immune system**	Yes	3(2.2)	1 (0.8)	0.78	*0*.*377*
	No	133(97.8)	119 (99.2)		
**Cardiac disease**	Yes	4(2.9)	4(3.3)	0.03	*0*.*857*
	No	132(97.1)	116(96.7)		

*Fisher’s Precise probabilities.

In the study, we examined the hospitalization reasons of two patient groups, encompassing induced 4 abortions, 178 childbirths(128 deliveries, 50 cesarean sections), 24 dysfunctional uterine bleeding, 25 hysteromyoma, 2 ovarian cysts, 4 perineal hematomas, 9 inflammatory diseases, 9 prolapse, and 1 miscarriage. Individuals with visual impairment or paralysis were excluded from the study.

We also examined the utilization of sedatives or analgesics among inpatients. 85 parturients were administered analgesics during labor, with ropivacaine (Qilu Pharmaceutical Co., Ltd.) being the primary agent utilized. 88 surgical inpatients (50 cesarean sections and 38 gynecologic surgeries), patient-controlled analgesia pumps were employed within 48 hours postoperatively. The analgesics administered via these pumps included butorphanol (Jiangsu Hengrui Pharmaceuticals Co.,ltd.) and dexmedetomidine (Cisen Pharmaceutical Co., Ltd.). 5 surgical inpatients fell while receiving analgesics.

When examining different periods, the number of falls exhibited variation (χ2 = 11.926, *P* = 0.003). The majority of falls occurred in the afternoon, with 64 (47.2%) falls happening between 12:00 and 18:00, followed by 39 (28.7%) falls between 06:00

### 4.2 Validity

#### MFS score

The MFS scores for the no-fallers ranged from 0 to 90, with a mean score of 31.71±17.156. Conversely, the MFS scores for the fallers ranged from 0 to 85, with a mean score of 54.18±21.06. Both groups exhibited skewed distributions (Z = 2.544, *P =* 0.000; Z = 2.126, *P =* 0.000). Upon comparing the scores between the two groups, the Fall group demonstrated significantly higher scores than the No-fall group (Z = 8.153, *P*<0.001).

#### ROC of MFS

**Fig 1:**
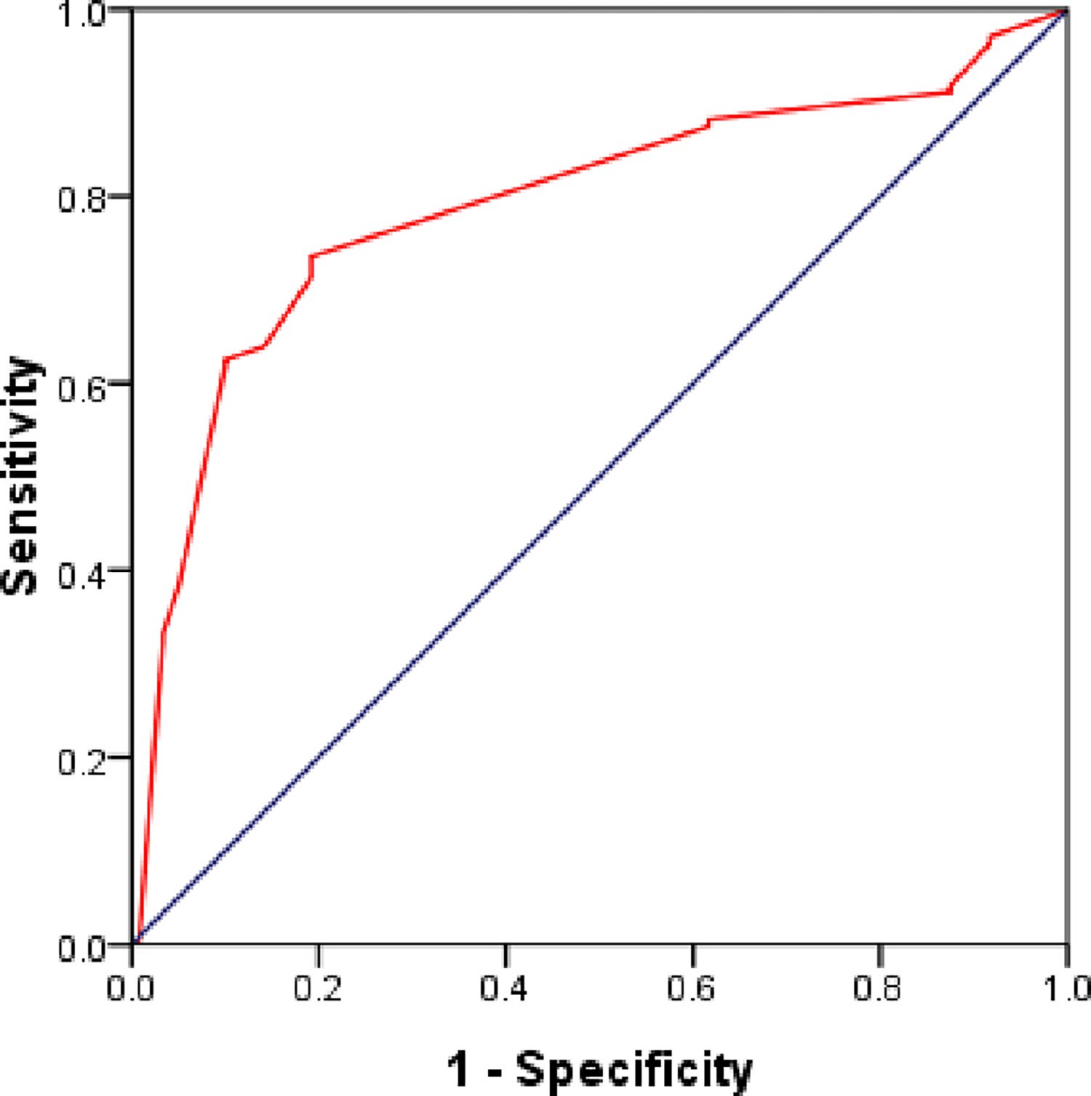
Receiver operating characteristic (ROC) curve. The receiver operating characteristic (ROC) curve with the area under the curve (AUC) of the MFS. The largest areal is 0.791±0.029.

Fig 1 shows the ROC curve of MFS. The AUC was 0.791±0.029 (AUC>0.5, *P =* 0.000) for the ROC curve, and the AUC 95% CI was (0.733, 0.848).

### Sensitivity, specificity, and Youden index

We conducted tests using 5-point intervals, and increased the number of tests in the cut-off interval corresponding to the peak and sub-peak of the Youden index, to avoid omission. As detailed in Table 2, the cut-off scores were systematically assessed within the range of 15 to 80. Sensitivity showed a gradual decrease, and specificity increased as the cut-off score increased. The Youden index exhibited a peak at the cut-off score of 37.5–40, followed by a secondary peak at 52.5–55 owing to the absence of scores ranging from 36-39/41-44/46-49 in the database. Subsequently, the Youden index decreased with further increases in cut-off scores. AUC fluctuated between 0.500 and 0.772. The most substantial AUC, reaching 0.772, was observed at the cut-off score of 40, with notable AUC values of 0.763 and 0.761 at cut-off scores of 55 and 45, respectively.

**Table 2:** Sensitivity, specificity, Youden index and AUC at different MFS cut-off scores.

Cut-off Score	Sensitivity	Specificity	Youden index	AUC	Standard error ^a^	95% Confidence Interval	
						Lower limit	Upper limit
15	0.963	0.083	0.046	0.523	0.036	0.452	0.594
20	0.912	0.125	0.162	0.518	0.036	0.447	0.589
25	0.882	0.383	0.264	0.633	0.035	0.564	0.702
30	0.875	0.383	0.258	0.629	0.035	0.560	0.698
35	0.875	0.383	0.258	0.629	0.035	0.560	0.698
37.5	0.735	0.808	0.543	0.772	0.030	0.712	0.831
39.5	0.735	0.808	0.543	0.772	0.030	0.712	0.831
**40**	**0.735**	**0.808**	**0.543**	**0.772**	**0.030**	**0.712**	**0.831**
42.5	0.713	0.808	0.521	0.761	0.031	0.712	0.831
**45**	**0.713**	**0.808**	**0.521**	**0.761**	**0.031**	**0.700**	**0.821**
47.5	0.640	0.858	0.498	0.749	0.031	0.688	0.810
**50**	**0.640**	**0.858**	**0.498**	**0.749**	**0.031**	**0.688**	**0.810**
52.5	0.625	0.900	0.525	0.763	0.030	0.703	0.822
**55**	**0.625**	**0.900**	**0.525**	**0.763**	**0.030**	**0.703**	**0.822**
60	0.618	0.900	0.518	0.759	0.031	0.699	0.819
65	0.382	0.950	0.332	0.666	0.034	0.600	0.732
70	0.331	0.967	0.298	0.649	0.034	0.582	0.716
75	0.324	0.967	0.291	0.645	0.034	0.578	0.712
80	0.007	0.992	-0.001	0.500	0.036	0.429	0.571

### 4.3 The optimal cut-off score for the MFS

As per the data presented in Table 3, a comparative analysis among cut-off scores of 40,45,50 and 55 reveals that the cut-off score of 40 yields superior results, evident in a higher Youden index (0.543), Kappa coefficient [(0.540, 95% confidence interval (95% CI) = 43.8%–64.3%], an acceptable sensitivity of 0.735 (95% CI = 59.8%–89.4%), fairly good specificity (0.808, 95% CI = 65.6%–98.6%), Positive Predictive Value (PPV) of 0.813 (95% CI = 66.2%–98.9%), Negative Predictive Value (NPV) of 0.729 (95% CI = 59.1%–89.0%), and an overall accuracy of 0.770 (95% CI = 66.6%–88.5%).

**Table 3:** Comparison of MFS Performance with cut-off scores 40,45, 50and 55.

Cut-off Scores	AUC	Sensitivity	Specificity	Youden Index	PPV	NPV	Accuracy	Kappa
≥40	0.772	0.735	0.808	0.543	0.813	0.729	0.770	0.540*
≥45	0.761	0.713	0.808	0.521	0.808	0.713	0.758	0.540*
≥50	0.749	0.640	0.858	0.498	0.837	0.678	0.742	0.490*
≥55	0.763	0.625	0.900	0.525	0.876	0.679	0.754	0.515*

* Statistically significant(Z = 8.688,8.345, 8.090,8.641, *P<*0.001).

## 5. Discussion

This study represents the first application of the MFS in gynecological and obstetric wards in China, and direct comparisons with studies conducted in community and rehabilitation wards are currently unavailable.

### 5.1 Factors affecting falls in the obstetrics and gynecology ward

Previous research consistently highlights the influence of physical impairments and weakness associated with aging, contributing to an elevated risk of falling and subsequent injuries [12,13]. However, our investigation reveals no discernible distinctions in the demographic characteristics of patients between the fall and non-fall groups in gynecological and obstetric wards. This finding contrasts with numerous studies conducted in acute wards, reporting variations in falls based on gender and age [1,6]. Consistent with previous studies [8,15], falls have been associated with surgery. We also observed five cases of falls among inpatients who underwent surgery and used pain-relief medications afterward. However, due to sample size limitations, we were unable to distinguish between the effects of surgery and pain-relief medications on falls.

While insomnia and activity levels did not exhibit a notable association. This may be attributed to the distinctive characteristics of women in gynecology and obstetrics wards, predominantly young and fertile, displaying ample physical strength, unrestricted mobility, better sleep quality, and higher activity levels. This observation may explain the absence of age, activity level, and insomnia as discernible fall risk factors in obstetrics and gynecology.

### 5.2 The incidence of falls in the obstetrics and gynecology wards

In this study, the observed fall rates in gynecology and obstetrics wards were notably lower at 0.504 per 1000 patient days compared to medical units in Boston and New York City using the Patient-Centered Fall-Prevention Tool Kit, where rates ranged from 2.92 to 2.49 per 1000 patient days [14]. Furthermore, these rates were also lower than those reported in a surgical unit in the southeastern United States, which experienced an average monthly preintervention fall rate of 8.67 falls per 1000 patient days, decreasing to 5.07 falls postintervention [15]. The lower decline rate in the obstetrics and gynecology wards in China may be influenced not only by the factors mentioned above but also by the practices of Chinese nursing staff. Given that most Chinese patients receive one-on-one care from their families, or even more, timely assistance from caregivers could play a mitigating role in reducing the risk of falls.

### 5.3 Implications from fall events

An analysis of fall records in this study revealed a concentration of falls between 12:00 and 18:00. This temporal trend can be explained by the fact that most medical treatments are typically administered in the morning, potentially elevating fall risk as patients engage in more autonomous activities during the afternoon. This observation underscores the importance for nurses to be particularly vigilant during the patients’ more active periods, emphasizing the need for enhanced fall prevention measures.

### 5.4 The application of MFS in obstetrics and gynecology wards demonstrates good effectiveness

Through the computation of key metrics, including sensitivity, specificity, positive predictive value (PPV), negative predictive value (NPV), and accuracy, we assessed the discriminatory capability of the scale. The findings indicate that the MFS exhibits a commendable ability to differentiate fall risk among gynecological and obstetric patients, with an AUC area of 0.772. This performance compares favorably to previous studies, such as Hye-Mi Noh’s investigation, which reported an AUC area of 0.615 in elderly inpatients [16], and Sikha Bagui’s research with an AUC area of 0.5967 conducted at a community-owned hospital [17]. This implies that the MFS may offer enhanced predictive accuracy in the context of gynecological and obstetric patients compared to its performance in other settings, showcasing its utility as a reliable tool for assessing fall risk in these specialized wards.

### 5.5. The best optimal cut-off score of MFS

We observed that the cut-off score of 40 yields the largest AUC area and propose that the optimal cut-off score for gynecological and obstetric inpatients is 40 points. Although the AUC area for a cut-off score of 40 (0.772) is marginally smaller than the largest AUC area 0.791, it still suggests that the MFS demonstrates competency in predicting a patient’s risk of falling, given that the AUC exceeds 0.7. This aligns with the findings of Sikha Bagui [17], who conducted a study in a community-owned hospital and recommended adjusting the cut-off value to 40 points (Sensitivity 63.77%, Specificity 50.44%). However, our suggestion differs from Morse’s earlier proposal of a low-risk cut-off score of 25 and a high-risk cut-off score of 51 [7], Hye-Mi Noh’s recommended cut-off score of 45 [16], and Baek’s suggestion of 51 points as the optimal threshold [18].

## 6. Advantages and limitations

Despite the above findings, this study has limitations. Firstly, the use of a purposive sample and a retrospective design imposes constraints on the generalizability of the study. Terry P Haines [19] reported that Design-related bias in evaluations of tool predictive accuracy could lead to overoptimistic results where Retrospective evaluations had significantly higher Youden Indices.

Secondly, the MFS was not subjected to a comparative analysis with other tools for assessing fall risk. Therefore, future research employing a prospective design is imperative to validate the applicability of MFS in gynecological and obstetric wards.

## 7. Conclusions

This study demonstrates the efficacy of the MFS in predicting fall risk within gynecological and obstetric wards. The findings highlight that, specifically for gynecological and obstetric inpatients, the MFS demonstrates good effectiveness and exhibits optimal performance when applied with a cut-off score of 40.
